# Non-dopaminergic Alterations in Depression-Like FSL Rats in Experimental Parkinsonism and L-DOPA Responses

**DOI:** 10.3389/fphar.2020.00304

**Published:** 2020-03-20

**Authors:** Nicoletta Schintu, Xiaoqun Zhang, Nikolas Stroth, Aleksander A. Mathé, Per E. Andrén, Per Svenningsson

**Affiliations:** ^1^Department of Clinical Neuroscience, Karolinska University Hospital, Karolinska Institutet, Stockholm, Sweden; ^2^Medical Mass Spectrometry Imaging, Department of Pharmaceutical Biosciences, Uppsala University, Uppsala, Sweden; ^3^Science for Life Laboratory, Uppsala University, Uppsala, Sweden

**Keywords:** flinders sensitive line, Parkinson’s disease, L-DOPA, dyskinesia, tremor, tamalin, GRASP

## Abstract

Depression is a common comorbid condition in Parkinson’s disease (PD). Patients with depression have a two-fold increased risk to develop PD. Further, depression symptoms often precede motor symptoms in PD and are frequent at all stages of the disease. However, the influence of a depressive state on the responses to antiparkinson treatments is largely unknown. In this study, the genetically inbred depression-like flinders sensitive line (FSL) rats and control flinders resistant line (FRL) rats were studied in models of experimental parkinsonism. FSL rats showed a potentiated tremorgenic response to tacrine, a cholinesterase inhibitor used experimentally to induce 6 Hz resting tremor reminiscent of parkinsonian tremor. We also studied rats lesioned with 6-OHDA to induce hemiparkinsonism. No baseline differences in dopaminergic response to acute apomorphine or L-DOPA was found. However, following chronic treatment with L-DOPA, FRL rats developed sensitization of turning and abnormal involuntary movements (AIMs); these effects were counteracted by the anti-dyskinetic 5-HT_1__A_ agonist/D_2_ partial agonist sarizotan. In contrast, FSL rats did not develop sensitization of turning and only minor AIMs in response to L-DOPA treatment. The roles of several non-dopamine systems underlying this discrepancy were studied. Unexpectedly, no differences of opioid neuropeptides or serotonin markers were found between FRL and FSL rats. The marked behavioral difference between the FRL and FSL rats was paralleled with the striatal expression of the established marker, c-fos, but also the GABAergic transporter (vGAT), and a hitherto unknown marker, tamalin, that is known to regulate mGluR5 receptor function and postsynaptic organization. This study demonstrates that behavioral and transcriptional responses of non-dopaminergic systems to experimental parkinsonism and L-DOPA are modified in a genetic rat model of depression.

## Introduction

Parkinson’s disease (PD) is the second most common neurodegenerative disorder and is diagnosed based on the presence of bradykinesia, rigidity and tremor (Kalia and Lang, 2015). PD patients often have comorbid depression that may precede the onset of motor signs, but is frequently present at all stages of the disease (Aarsland et al., 2012). When comparing PD patients to patients suffering from other chronic motor disabilities, parkinsonian individuals score higher in rating scales for depression (Robins, 1976).Thus, depression symptoms appear to be a part of PD pathology and not only a reaction to the somatic disease manifestations. In fact, unipolar depressed patients have a two-fold increased risk of being diagnosed with PD when compared to other groups with chronic illness that also need continuous treatment (Nilsson et al., 2001; Leentjens et al., 2003). Despite the clinical importance, few preclinical or clinical studies have examined how a depressive state affects the progression of PD or the response to antiparkinson treatments.

The flinders sensitive line (FSL) of rats is commonly used as a genetic animal model of depression with the flinders resistant line (FRL) serving as control (Overstreet et al., 2005). The FSL rat strain was established from Sprague Dawley rats through a selective breeding program for increased sensitivity to the cholinergic compound di-isopropyl fluorophosphates (DFP). FRL rats exhibit low sensitivity to DFP. FSL rats have good face validity and exhibit increased immobility in the forced swim test. The construct validity of this genetic model is reflected by the similarities with the human pathology affecting neurochemical systems including the serotonergic, dopaminergic and glutamatergic systems. In particular, studies have reported altered 5-HT_1__A_, D_1_, mGluR5 receptor levels in FSL rats (Bjørnebekk et al., 2007; Eriksson et al., 2012; Kovačević et al., 2012; Shrestha et al., 2014). The FSL rats also possess predictive validity, since all so far tested antidepressant therapies show antidepressant-like effects in these rats (Overstreet and Wegener, 2013).

L-DOPA is the most efficacious medication for the treatment of PD. Unfortunately, the therapeutic response to L-DOPA is wearing off over time, while abnormal involuntary movements (AIMs), such as dyskinesias, emerge as prominent side-effects in approximately 50% of all treated patients within 5–10 years (Obeso et al., 2000). Several molecular and cellular mechanisms involving non-dopaminergic systems have been proposed to explain the development of dyskinesias in PD (Cenci et al., 2011; Huot et al., 2013). One possibility is that in the advanced stage of dopaminergic cell loss, remaining serotonergic neurons in the basal ganglia complex can specifically take up L-DOPA and convert it to dopamine. In contrast to the normal situation, release of dopamine occurs in this setting when the serotonergic neurons are activated and dopamine acts as a false transmitter. Drugs that act on the 5-HT_1__A_ autoreceptor, which regulates the firing of serotonergic neurons, can antagonize L-DOPA-induced dyskinesia, as shown in preclinical and clinical studies (Bibbiani et al., 2001; Tomiyama et al., 2005; Carta et al., 2007; Svenningsson et al., 2015). Sarizotan is a 5-HT_1__A_ receptor agonist and dopamine D2-like partial agonist that reduces dyskinesia in PD animal models (Gregoire et al., 2009; Zhang et al., 2011a). Sarizotan has also been shown to have some anti-dyskinetic effect in PD patients (Bara-Jimenez et al., 2005; Goetz et al., 2007), but reduces also beneficial effects of L-DOPA (Goetz et al., 2007) and has not been approved for the treatment of dyskinesas. In addition to serotonin, studies have implicated the glutamatergic, cholinergic, opioidergic and GABAergic systems in L-DOPA-induced dyskinesias (Cenci et al., 2011).

The aim of the present study was to increase understanding of the influence of a depression-like genotype on experimental parkinsonism using tacrine-induced tremor and hemiparkinsonian FSL and FRL rats at baseline and upon chronic treatment with L-DOPA. A special emphasis was put on study non-dopaminergic alterations under these conditions.

## Materials and Methods

### Animals, Surgery and Pharmacological Treatment

Male FRL and FSL rats (290–390 g) were housed in air-conditioned rooms (12-h dark/light cycle) at 20°C and a humidity of 53%. Experiments were performed in agreement with the European Communities Council Directive of 24 November 1986 (86/609/EEC) on the ethical use of animals and were approved by the local ethical committee at Karolinska Institutet.

### Tacrine-Induced Jaw Movements

Flinders sensitive line (*n* = 12) and FRL (*n* = 10) rats were treated with tacrine (2.5 mg/kg, i.p., Sigma) to induce jaw movements which were then manually scored for 5 min after the 10-min habituation (Salamone et al., 1998).

### Unilateral 6-OHDA Lesion

**FIGURE 1:**
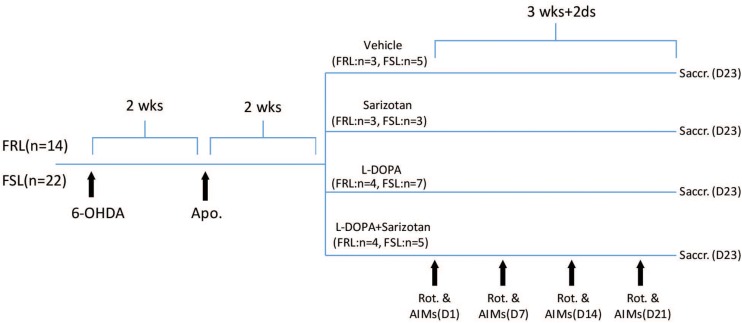
Schematic representation of the study design. FRL (*n* = 14) and FSL (*n* = 22) rats were injected with 6-OHDA (2.5 μl of a 5 mg/ml solution) into MFB of the right hemisphere. Two weeks after the unilateral 6-OHDA lesion, rats were injected with apomorphine (1 mg/kg, i.p.) and their contralateral rotations were measured to determine the degree of nigrostriatal denervation. Four weeks after surgery, rats were treated with saline (FRL, *n* = 3; FSL, *n* = 5), sarizotan (2.5 mg/kg, i.p.) (FRL, *n* = 3; FSL, *n* = 3), L-DOPA/benserazide (10/7.5 mg/kg, i.p.), alone (FRL, *n* = 4; FSL, *n* = 6) or in combination (FRL, *n* = 4; FSL, *n* = 5) once daily for 23 days. Rotational behavior and AIMs were measured on Day 1, Day 7, Day 14, and Day 21. Animals were sacrificed 30 min after the last drug administration.

As shown in Figure 1, another group of FRL (*n* = 14) and FSL (*n* = 22) rats were anesthetized with ketamine (100 mg/kg, i.p.; Intervet)/xylazine (5 mg/kg, i.p.; Bayer, Kiel, Germany), pretreated with desipramine (25 mg/kg, i.p.; Sigma, St Louis, MO, United States)/pargyline (5 mg/kg, i.p.; Sigma), placed in a stereotaxic instrument and injected with 6-OHDA (2.5 μl of a 5 mg/ml solution; Sigma) into the median forebrain bundle (MFB) of the right hemisphere (AP −2.8 mm, ML −2.0 mm, and V −9.0 mm). Two weeks after the unilateral 6-OHDA lesion, rats were injected with apomorphine (1 mg/kg, i.p.; Sigma) and their contralateral rotations were measured to determine the degree of nigrostriatal denervation. Only rats rotating >100 turns over 30 min were included in further experiments.

### Pharmacological Treatment and Behavioral Evaluation

Four weeks after surgery, rats were divided in groups according their rotation upon apomorphine so that they were similar in terms of anticipated dopamine lesion (Figure 1). They were treated with saline (FRL, *n* = 3; FSL, *n* = 5), sarizotan (2.5 mg/kg, i.p., Merck KGA, Darmstadt, Germany) (FRL, *n* = 3; FSL, *n* = 3), L-DOPA/benserazide (10/7.5 mg/kg, i.p., Sigma), alone (FRL, *n* = 4; FSL, *n* = 6) or in combination (FRL, *n* = 4; FSL, *n* = 5) once daily for 23 days. Once per week, rotational behavior and AIMs were measured. The number of contralateral rotations was manually counted for 2 h following drug administration. The incidence of AIMs was scored during turning behavior. AIMs were classified into three subtypes according to their topographic distribution, as previously described (Lundblad et al., 2002): axial, limb, and orolingual AIMs. Each AIM was quantified in a 5-min testing period every 10 min (starting 15 min after L-DOPA administration). The severity of each AIM subtype was assessed using scores from 0 to 4 (0: absent, 1: occasional, i.e., present less than 50% of the time; 2: frequent, i.e., present more than 50% of the time; 3: continuous, but interrupted by strong sensory stimuli, and 4: continuous, not interrupted by strong sensory stimuli).

Animals were sacrificed 30 min after the last drug administration. Brains were rapidly removed and frozen in dry ice-cooled isopentane. Some brains were cut in a cryostat at −20°C into 12 μm coronal sections and stored at −80°C until use.

### Explorative Microarray Gene Expression Analysis

Total RNA was extracted from the 6-OHDA-lesioned striatum of vehicle- or L-DOPA-treated FRL and FSL rats using Trizol (Sigma) followed by RNeasy (Qiagen, Hilden, Germany) clean-up according to manufacturer’s instructions. After quality control (Bioanalyzer), RNA samples were submitted to the Gene Expression Service Workflow of Roche NimbleGen, which included hybridization with a Rat Gene Expression 12 × 135K Array (Design Name 100718_Rat_HX12_expr), array scanning, data extraction and processing. Normalized gene expression data (“Normalized Calls”) provided by Roche NimbleGen were analyzed using ANAIS (Simon and Biot, 2010) via the “Norm genes” function. Genes were considered differentially expressed if their transcript abundance (scanned signal intensity) was ≥1.5-fold higher or lower in at least one experimental group compared to striatum of vehicle-treated FRL rats. The resulting list of 6040 genes was imported into Microsoft Excel, where only those genes with ANAIS-computed ANOVA *p*-values <0.05 were retained. At this stage, the list of candidates was subjected to manual annotation using NCBI’s Gene database, such that updated GeneIDs could be assigned to all candidates.

### *In situ* Hybridization Studies

Radioactive complementary probe for the vesicular GABAergic transporter (vGAT) and tamalin (also called GRP1-associated scaffold protein, GRASP) were made (Supplementary Table S1) and used along with probes for c-fos, enkephalin and dynorphin (Zhang et al., 2006, 2011b). Briefly, sections were pretreated with 4% paraformaldehyde for 5 min at room temperature, rinsed twice in 4 × sodium chloride–sodium citrate (SSC) buffer and placed into 0.25% acetic anhydride in 0.1 M triethanolamine/4 × SSC (pH 8) for 10 min at room temperature. After dehydration in graded alcohols, the sections were hybridized overnight at 55°C with 10^6^ c.p.m. of ^35^S-labeled probe in 50 μl of hybridization solution (20 mM Tris–HCl/1 mM EDTA/300 mM NaCl/50% formamide/10% dextran sulfate/1× Denhardt’s solution/250 g/ml yeast tRNA/100 g/ml salmon sperm DNA/0.1% SDS/0.1% sodium thiosulphate). The slides were washed in 4 × SSC (5 min, four times), RNAse A (20 g/ml) (20 min, at 37°C), 2 × SSC (5 min, twice), 1 × SSC (5 min), 0.5 × SSC (5 min) at room temperature, and rinsed in 0.1 × SSC at 65°C (30 min, twice), before being dehydrated in graded alcohols. The slides were then exposed to X-ray films for 5–21 days.

### Autoradiographic Studies

The radioligands [^125^I] RTI-55 (serotonin and dopamine transporter binding) and [^125^I] MPPI [4-(2′methoxyphenyl)-1-2′[2′-pyridinyl-)-iodo-benzomido] piperazine (5-HT_1__A_ binding) were purchased from Perkin Elmer Life Sciences Inc. (Boston, MA, United States).

Section for detection of DAT (dopamine transporter) and serotonin transporter (SERT) (serotonin transporter) were preincubated in 50 mM Tris–HCl/120 mM NaCl (pH 7.5) for 20 min, incubated for 1 h in the same buffer supplemented with 50 pM [^125^I] RTI-55 in the presence of either 1 μM fluoxetine (selective serotonin reuptake inhibitor; Sigma) to label the DAT or 1 μM nomifensine (dopamine reuptake inhibitor; Sigma) to label the SERT. For non-specific binding, 100 μM nomifensine or 100 μM fluoxetine were added to the assay. The slides were washed 2 × 10 s in ice-cold binding buffer, rapidly dipped in deionized water and dried.

Sections for detection of 5-HT_1__A_ receptors were preincubated with 50 mM Tris–HCl containing 2 mM MgCl_2_ (pH 7.4) for 30 min, incubated for 2 h in the same buffer together with 0.01 nM [^125^I] MPPI. For non-specific binding, 10 μM serotonin was added. The slides were washed 2 × 15 min in ice-cold binding buffer, quickly dipped in deionized water and dried. The sections were then exposed to Kodak BioMax MR films (Sigma) for 2 days.

### Data Analysis of Autoradiograms From Ligand-Binding or *in situ* Hybridization Experiments

For ligand-binding and *in situ* hybridization experiments, autoradiograms were digitized using a Dia-Scanner (Epson Perfection 4870 PHOTO). Optical density values were measured using Image J. For the analysis of the striatum, measurements in the 6-OHDA-lesioned hemisphere are given as ratios of the corresponding area from the intact hemisphere. For analysis in the raphe nuclei, data are expressed as percentage of the vehicle-treated FRL rats group.

### Experiment With Sprague Dawley Rats

Male Sprague Dawley rats have been injected with 6-OHDA following exactly the same protocol described for the FSL and FRL experiment. After injecting the rats with apomorphine (1 mg/kg, i.p.), to evaluate the efficacy of the 6-OHDA lesioning, rats were treated with vehicle or L-DOPA/benserazide (10/7.5 mg/kg, i.p.) for 1 day (acute L-DOPA) or 4 weeks (chronic L-DOPA). 30 min after the last injection, brains have been removed and stored as described above for FRL and FSL rats.

### Statistics

Behavioral and biochemical results were analyzed with two-way ANOVA followed by Fisher *post hoc* test for comparison between experimental groups. In the analysis of the contralateral turning behavior, time and treatment were used as independent factors; genotype and treatment were the independent factors for the analysis of AIMs and *in situ* hybridization data. In the experiment with Sprague-Dawley rats treated with saline, acute or chronic L-DOPA, one-way ANOVA was used followed by Fisher *post hoc* test. In the tacrine-induced jaw movement’s experiment, where only two groups were compared, Student *t*-test was used for statistical analysis.

## Results

### Tacrine-Induced Jaw Movements

**FIGURE 2:**
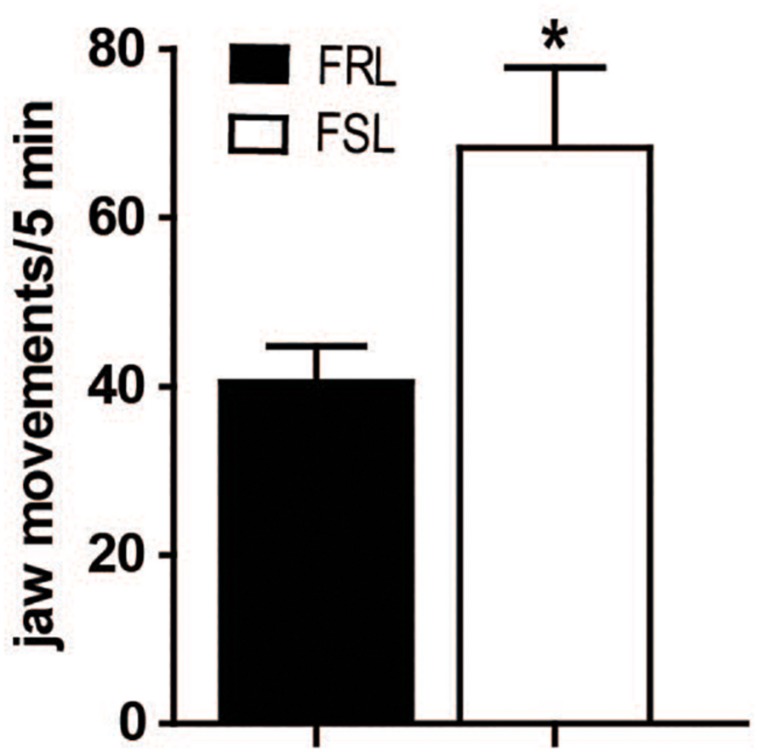
FSL rats showed increased tacrine-induced jaw movements. Effect of tacrine (2.5 mg/kg, i.p.) on jaw movement in FRL and FSL rats. Data represent mean ± SEM for 10–12 animals per group. *p < 0.01 vs FRL group, accordingly to student *t*-test.

Tremulous jaw movements induced by acute administration of the cholinesterase inhibitor tacrine (2.5 mg/kg, i.p.) were more frequent in FSL than in FRL rats (*p* < 0.05, Figure 2). This result indicates that movement responses reminiscent of parkinsoniam 6 Hz tremor are exaggerated in FSL compared to FRL rats.

### Efficacy of Unilateral 6-OHDA Lesion

**FIGURE 3:**
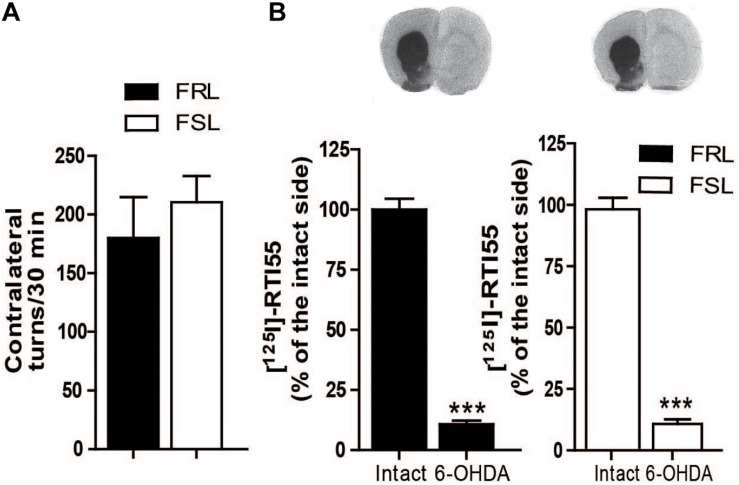
Efficacy of 6-OHDA lesion is shown by apomorphine-induced contralateral turning behavior and dopamine transporter binding. **(A)** Shows the number of contralateral turns in FRL and FSL rats treated with apomorphine (1 mg/kg, i.p.). **(B)** Shows autoradiograms and histograms of [^125^I] RTI-55 binding for analysis of the dopamine transporter in FRL and FSL rats. Data were generated as optical density measures. Data represent mean ± SEM of the values expressed as a percentage of the intact hemisphere for three to seven animals per group. ***p < 0.001 vs correspondent intact hemisphere.

6-OHDA-induced lesioning of the MFB induces a near-complete degeneration of the nigrostriatal dopamine neurons. Supersensitization of dopaminergic receptors in the lesioned hemisphere results in induction of contralateral turning behavior after administration of dopaminergic drugs. Thus, to assess the degree of dopaminergic lesion, we measured the number of contralateral rotations after administration of the dopaminergic D_1_/D_2_ receptors agonist apomorphine (1 mg/kg, i.p.). No difference was found between FRL and FSL rats (Figure 3A). Likewise, [125I]RTI-55 autoradiography of DAT in striatum post-mortem did not detect any change in the degree of the dopaminergic lesioning between FRL and FSL rats (Figure 3B).

### Contralateral Turning Behavior and Abnormal Involuntary Movements After L-DOPA and Sarizotan Treatment

Chronic treatment with L-DOPA elicits locomotion and also abnormal motor responses such as sensitization of contralateral turning behavior and AIMs in unilaterally 6-OHDA-lesioned rodents (Lundblad et al., 2002).

**FIGURE 4:**
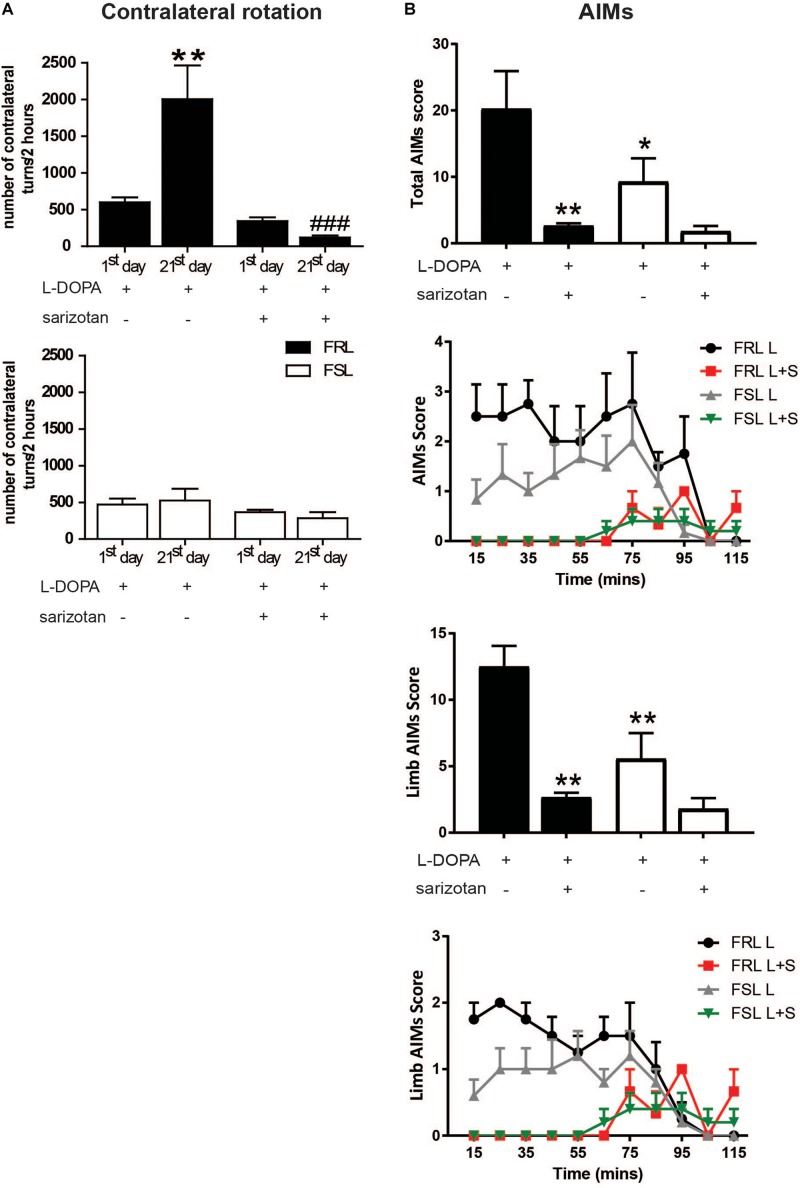
Unilaterally 6-OHDA-lesioned FSL and FRL rats respond differently to chronic treatment with L-DOPA and sarizotan/L-DOPA in terms of turning behavior and abnormal involuntary movement. Effect of chronic treatment with L-DOPA (10 mg/kg, i.p.) and sarizotan/L-DOPA (2.5/10/mg/kg, i.p.) on **(A)** the number of contralateral turns at the 1st and 21st day of treatment and (B) Total sum of each AIMs scores, limb AIMs score and the time course at the 21st day of treatment in FRL and FSL rats. Data represent mean ± SEM for four to seven animals per group. **(A)** **p < 0.01 vs FRL group at the 1st day of L-DOPA treatment, ^###^*p* < 0.001 vs FRL group at the 21st day of L-DOPA treatment. **(B)** *p < 0.05, **p < 0.01 vs FRL group treated with chronic L-DOPA accordingly to two-way ANOVA followed by Fisher *post hoc* test.

The first administration of L-DOPA induced similar contralateral turning behavior in both FRL and FSL rats (Figure 4A). However, the response to chronic L-DOPA treatment differed markedly between the two genotypes. In FRL rats, significant effects of treatment (*F*_1_,_12_ = 15.18; *p* < 0.01), time (*F*_1_,_12_ = 4.64; *p* < 0.05) and their interaction (*F*_1_,_12_ = 8.79; *p* < 0.05) were found. *Post hoc* test showed a significant increase in the number of rotations observed after chronic treatment with L-DOPA compared to the first administration (*p* < 0.001, Figure 4A). In contrast, in FSL rats, chronic L-DOPA treatment did not induce a significant change in the number of rotations observed, compared to acute administration (Figure 4A).

Chronic treatment with L-DOPA induced AIMs in both strains of rats (Figure 4B). However, there were significant effects of treatment (*F*_1_,_15_ = 37.85; *p* < 0.005). *Post hoc* test showed that FRL rats treated with L-DOPA spent more time in AIMs than FSL rats (*p* < 0.05). For the limb AIMs score, there were significant effects of treatment (*F*_1_,_15_ = 41.07; *p* < 0.005) and genotype (*F*_1_,_15_ = 13.48; *p* < 0.05). *Post hoc* test showed that FRL rats treated with L-DOPA spent more time in limb AIMs than FSL rats (*p* < 0.01).

The first co-administration of sarizotan/L-DOPA induced similar contralateral rotations in FRL and FSL rats (Figure 4A). Furthermore, chronic co-administration of sarizotan/L-DOPA did not induce any change in contralateral rotations compared to the first day of treatment. Accordingly, the number of contralateral turns in chronically treated sarizotan/L-DOPA FRL rats was significantly lower (*p* < 0.001) than in chronically L-DOPA-treated FRL (Figure 4A). No difference in the number of contralateral turns was found between sarizotan/L-DOPA- and L-DOPA-treated FSL rats. Likewise, co-administration with sarizotan significantly (*p* < 0.01) reduced L-DOPA-induced AIMs in FRL, but not in FSL, rats (Figure 4B).

### Biochemical Mechanisms Underlying Dyskinesia

#### c-fos

**FIGURE 5:**
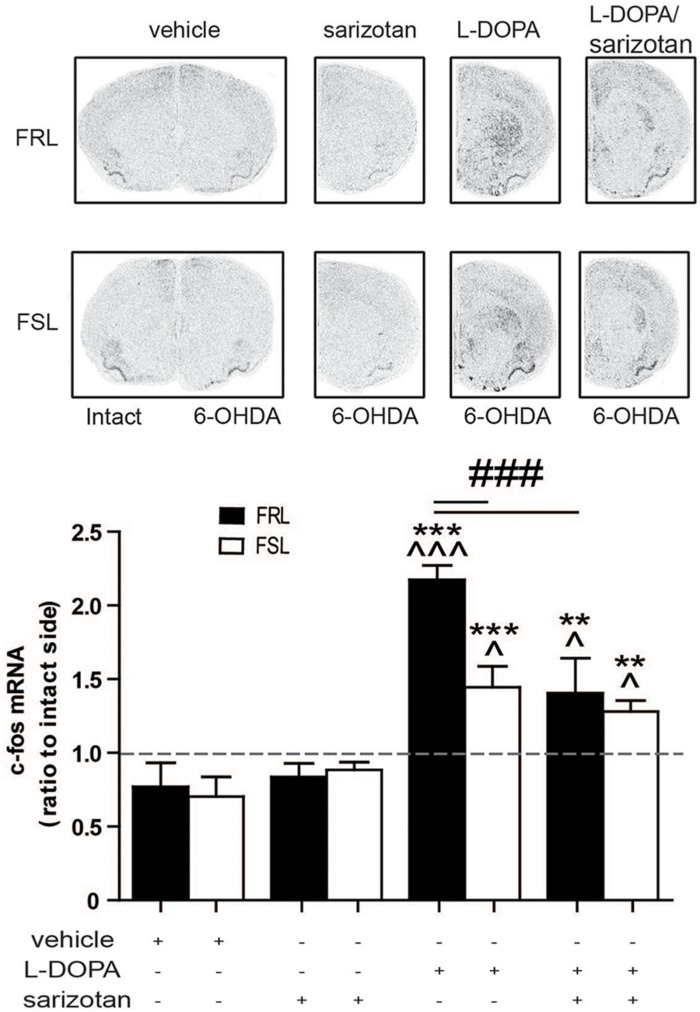
Regulation of c-fos mRNA levels in unilaterally 6-OHDA-lesioned FRL and FSL rats treated in response to chronic treatment with L-DOPA and sarizotan/L-DOPA. Autoradiograms and histograms of *in situ* hybridization experiment against c-fos mRNA in the striatum of FRL and FSL rats chronically treated with vehicle, sarizotan (2.5 mg/kg, i.p.), L-DOPA (10 mg/kg, i.p.) and sarizotan/L-DOPA (2.5/10 mg/kg, i.p.). Data represent mean ± SEM of the ratio between the lesioned and the intact hemisphere for three to seven animals per group. **p < 0.01, ***p < 0.001 vs correspondent vehicle-treated group; ^*p* < 0.05, ^^^*p* < 0.001 vs correspondent sarizotan-treated group, ^###^*p* < 0.001 vs indicated groups, accordingly to two-way ANOVA followed by Fisher *post hoc* test.

c-fos is an inducible transcription factor that is rapidly transcribed in response to a variety of stimuli often related to increased neuronal activity (Morgan and Curran, 1989). c-fos and related Fos/Jun members, particularly deltaFosB protein, have been linked to the development of AIMs in several animal models of PD (Cenci et al., 2011). In our model of comorbid depression and PD, genotype (*F*_1_,_25_ = 4.80; *p* < 0.05), treatment (*F*_3_,_25_ = 19.76; *p* < 0.001) and their interaction (*F*_3_,_25_ = 3.14; *p* < 0.05) had significant effects on c-fos expression. *Post hoc* test showed that chronic treatment with L-DOPA significantly increased c-fos mRNA compared to vehicle- and sarizotan-treated rats, in both FRL and FSL groups (*p* < 0.001; Figure 5). However, c-fos mRNA in L-DOPA-treated FSL rats was significantly lower than in FRL rats (*p* < 0.001; Figure 5). c-fos mRNA in sarizotan/L-DOPA-treated FRL rats was significantly lower than in the L-DOPA treated group (*p* < 0.001; Figure 5), whereas no significant difference between these treatments was found in FSL rats.

#### Opioid Neuropeptides

Increased striatal levels of enkephalin and dynorphin are associated with the development of dyskinesia in parkinsonian animals treated with L-DOPA (Sgroi and Tonini, 2018).

**FIGURE 6:**
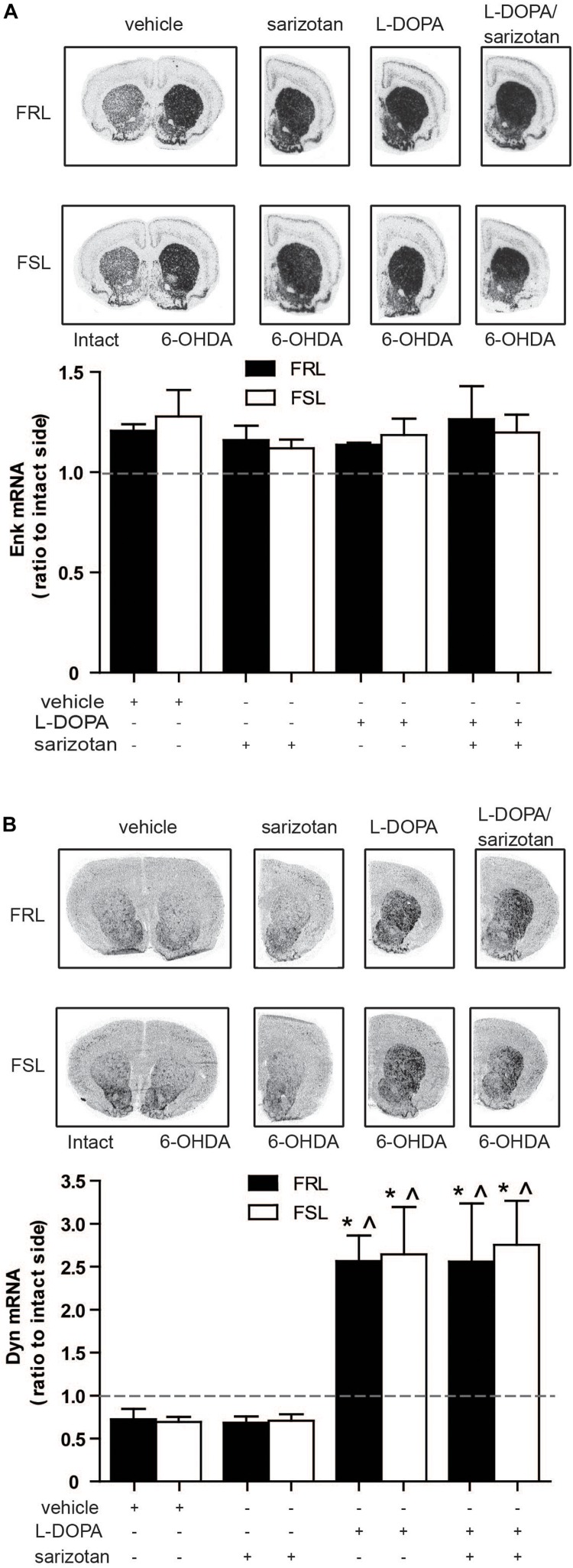
Effect of chronic treatment with L-DOPA and sarizotan/L-DOPA on the enkephalin and dynorphin mRNA levels in 6-OHDA-lesioned FRL and FSL rat. Autoradiograms and histograms of *in situ* hybridization experiment against **(A)** enkephalin and **(B)** dynorphin mRNAs in the striatum of FRL and FSL rats chronically treated with vehicle, sarizotan (2.5 mg/kg, i.p.), L-DOPA (10 mg/kg, i.p.) and sarizotan/L-DOPA (2.5/10 mg/kg, i.p.). Data represent mean ± SEM of the ratio between the lesioned and the intact hemisphere for three to seven animals per group. **(B)** *p < 0.05 vs correspondent vehicle-treated group; ^*p* < 0.05 vs correspondent sarizotan-treated group, accordingly to two-way ANOVA followed by Fisher *post hoc* test.

Surprisingly, neither treatment nor genotype (Figure 6A) modified enkephalin mRNA. Dynorphin mRNA revealed a significant difference for treatment (*F*_3_,_25_ = 11.76; *p* < 0.001). *Post hoc* test showed that dynorphin mRNA was significantly increased (*p* < 0.05) in the L-DOPA and sarizotan/L-DOPA groups compared with vehicle and sarizotan alone in both genotypes (Figure 6B).

#### Serotonergic System

As mentioned above, there is accumulating evidence for the involvement of serotonergic neurons in the development of AIMs. In particular, stimulation of 5-HT_1__A_ autoreceptors located at the cell body reduces the firing of serotonergic neurons and thus the release of “false” dopamine formed from L-DOPA.

**FIGURE 7:**
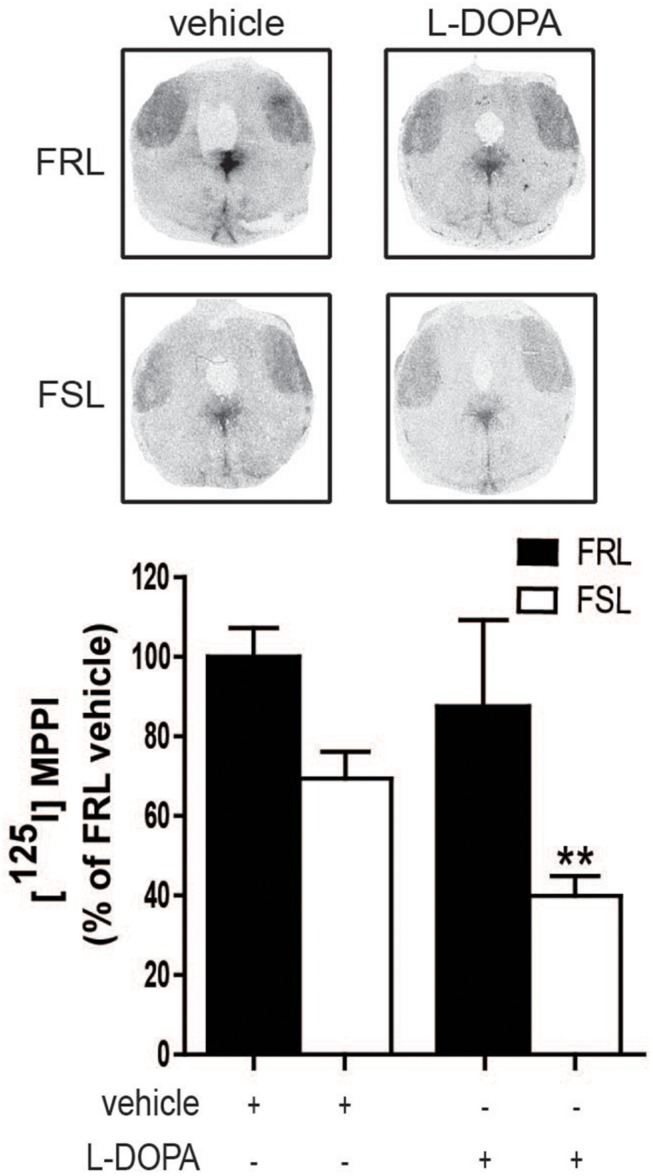
5-HT_1__A_ levels in the raphe nuclei of 6-OHDA-lesioned FRL and FSL rat chronically treated with L-DOPA. Autoradiograms and histograms of [^125^I] MPPI binding experiment in the raphe nuclei of FRL and FSL rats chronically treated with vehicle and L-DOPA (10 mg/kg, i.p.). Data were generated as optical density measures. Data represent mean ± SEM of the percentage of the vehicle-treated FRL group, for three to seven animals per group. **p < 0.001 vs L-DOPA-treated FRL group, accordingly to two-way ANOVA followed by Fisher *post hoc* test.

**FIGURE 8:**
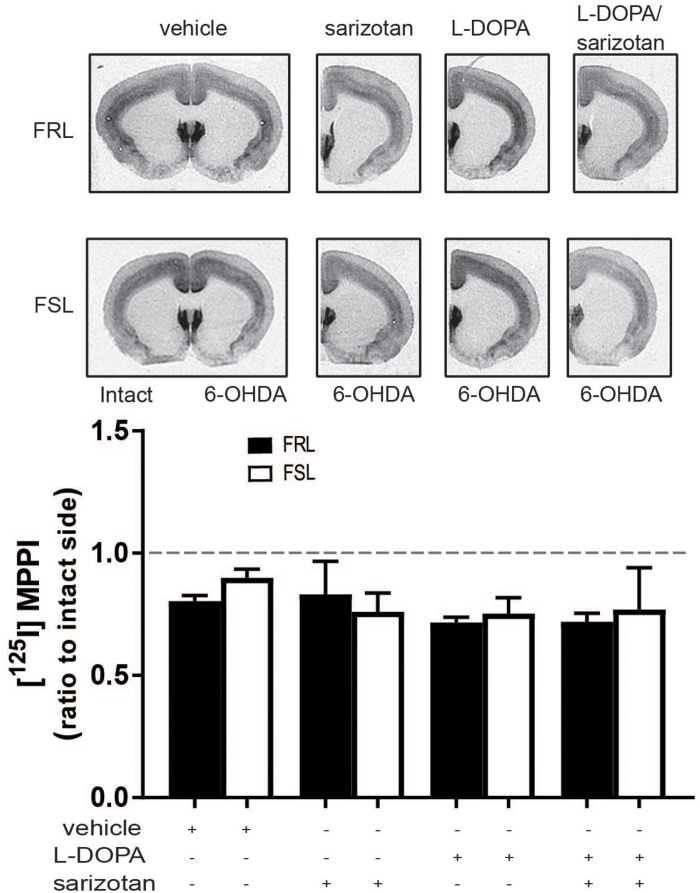
5-HT_1__A_ levels in the striatum of 6-OHDA-lesioned FRL and FSL rat chronically treated with L-DOPA. Autoradiograms and histograms of [^125^I] MPPI binding experiment in the striatum of FRL and FSL rats chronically treated with vehicle, sarizotan (2.5 mg/kg, i.p.), L-DOPA (10 mg/kg, i.p.) and sarizotan/L-DOPA (2.5/10 mg/kg, i.p.). Data were generated as optical density measures. Data represent mean ± SEM of the ratio between the lesioned and the intact hemisphere for three to seven animals per group.

In our study, [^125^I]MPPI binding to 5-HT_1__A_ receptor in the raphe nuclei of vehicle and L-DOPA treated rats revealed a significant effect of genotype (*F*_1_,_14_ = 11.60; *p* < 0.01) with lower levels in FSL than in FRL rats. In L-DOPA treated rats, levels of [^125^I]MPPI were lower in FSL compared to the FRL group (*post hoc* test *p* < 0.01; Figure 7). The decreased level of 5-HT_1__A_ receptor in the raphe nuclei unlikely explains the low dyskinetic potential of L-DOPA in FSL rats, but may relate to the fact that sarizotan is less antidyskinetic in FSL compared to FRL rats. There was no significant differences in [^125^I]MPPI binding in the striatum between hemiparkinsonian FSL and FRL rats (Figure 8).

**FIGURE 9:**
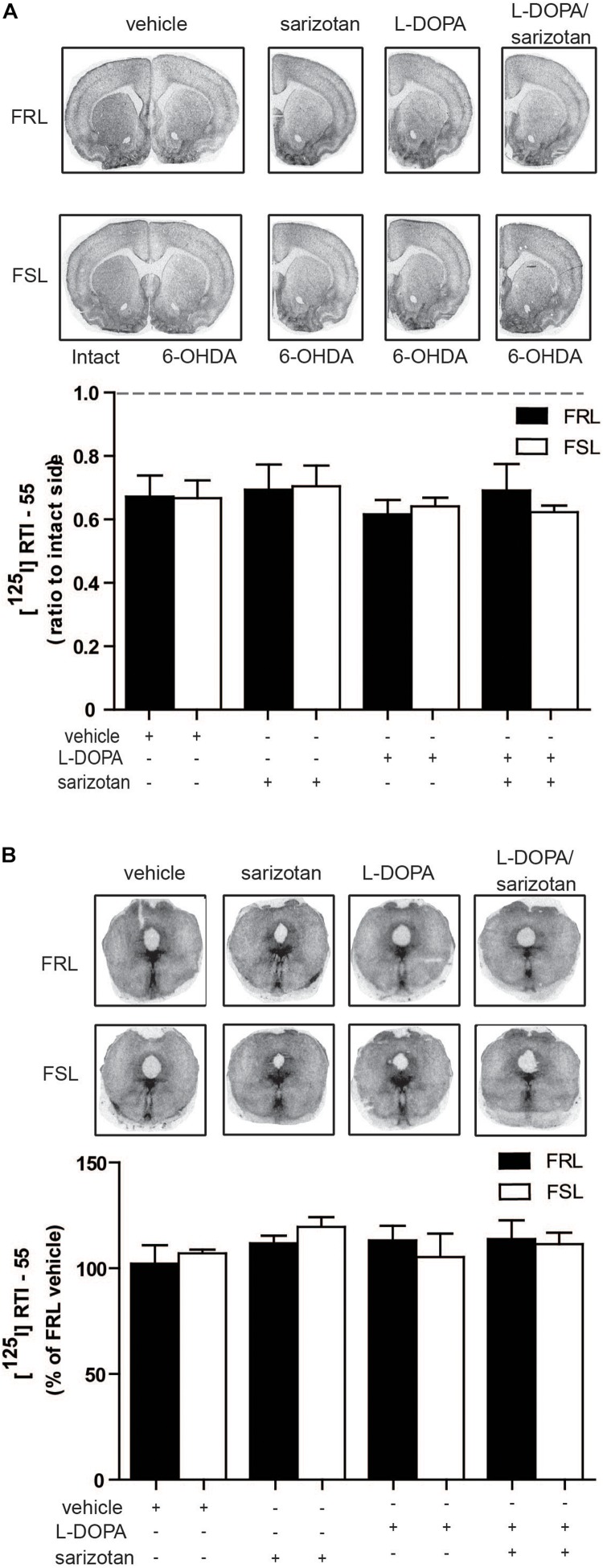
Serotonin transporter levels in the striatum and raphe nuclei of 6-OHDA-lesioned FRL and FSL rat chronically treated with L-DOPA and sarizotan/L-DOPA. Autoradiograms and histograms of [^125^I] RTI-55 binding experiments in **(A)** the striatum and **(B)** raphe nuclei of FRL and FSL rats chronically treated with vehicle, sarizotan (2.5 mg/kg, i.p.), L-DOPA (10 mg/kg, i.p.) and sarizotan/L-DOPA (2.5/10 mg/kg, i.p.). Data were generated as optical density measures. Data represent mean ± SEM of **(A)** the ratio between the lesioned and the intact hemisphere and **(B)** the percentage of the vehicle-treated FRL group, for three to seven animals per group, accordingly to two-way ANOVA followed by Fisher *post hoc* test.

Since a correlation between striatal SERT and severity of AIMs has been reported in 6-OHDA-lesioned rats, MPTP-treated macaque monkeys and in PD patients (Rylander et al., 2010), we also performed radioligand binding experiments for SERT. However, no significant differences in [^125^I]RTI-55 binding in the striatum or raphe nuclei between hemiparkinsonian FSL and FRL rats were found (Figures 9A,B).

#### Explorative Microarray Study

The aforementioned analyses of opioid neuropeptides and serotonin systems previously linked to AIMs did not yield correlates to the behavioral findings in our model of comorbid depression and PD. Therefore we decided to perform an explorative microarray analysis with 6-OHDA-lesioned striata collected from FRL and FSL rats (three animals per group) chronically treated with vehicle or L-DOPA. After selecting only genes that were 1.5-fold statistically different from the vehicle-treated FRL group, a list of 856 genes was obtained. Interestingly, in the chronic L-DOPA-treated FRL rats, 224 genes were found to be upregulated compared to the vehicle-treated FRL group, while only 68 were found in the L-DOPA-treated FSL group, indicating a generally lower response in the depression-like genotype (Supplementary Tables S2 and S3). The list of candidate genes was manually investigated looking for genes potentially linked to dyskinesia. c-fos was found to be in the list and already validated by our previous *in situ* hybridization result (Figure 4). Some other genes were chosen based on a possible correlation with dyskinesias for further validation by *in situ* hybridization.

#### Vesicular GABA Transporter, Previously Known to Correlated With AIMs

**FIGURE 10:**
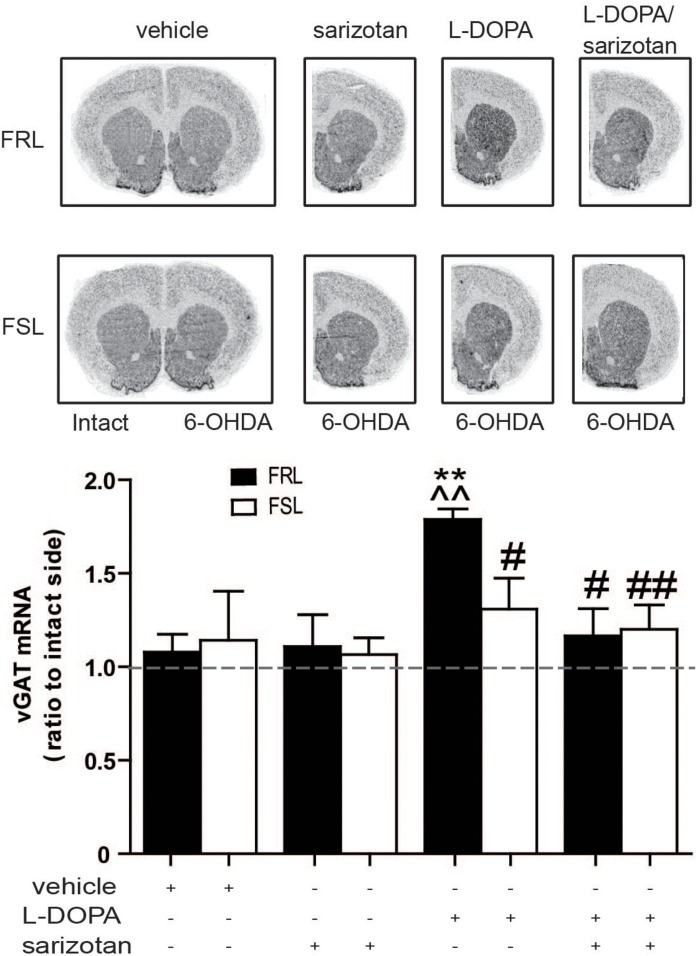
Different effect of chronic treatment with L-DOPA and sarizotan/L-DOPA on the vGAT mRNA levels in unilaterally 6-OHDA-lesioned FRL and FSL rats. Autoradiograms and histograms of *in situ* hybridization experiment against vGAT mRNA in the striatum of FRL and FSL rats chronically treated with vehicle, sarizotan (2.5 mg/kg, i.p.), L-DOPA (10 mg/kg, i.p.) and sarizotan/L-DOPA (2.5/10 mg/kg, i.p.). Data represent mean ± SEM of the ratio between the lesioned and the intact hemisphere for three to seven animals per group. **p < 0.01 vs correspondent vehicle-treated group; ^^*p* < 0.01 vs correspondent sarizotan-treated group, ^#^*p* < 0.05, ^##^*p* < 0.01 vs FRL group chronically treated with L-DOPA, accordingly to two-way ANOVA followed by Fisher *post hoc* test.

GABA-related genes have previously been linked with AIMs (Wang et al., 2007). Among the genes found via microarray analysis, vGAT (Slc32a1) expression correlated well with the behavioral data. *In situ* hybridization studies revealed a significant effect of treatment (*F*_3_,_25_ = 3.72; *p* < 0.05). *Post hoc* test showed that chronic treatment with L-DOPA increased (*p* < 0.01) vGAT mRNA in FRL but not FSL rats when compared to vehicle- or sarizotan-treated groups (Figure 10). FRL rats treated with sarizotan/L-DOPA showed a significantly lower vGAT mRNA level than FRL rats treated with L-DOPA (*p* < 0.05; Figure 10).

#### Tamalin, Previously Unknown to Correlate With AIMs

**FIGURE 11:**
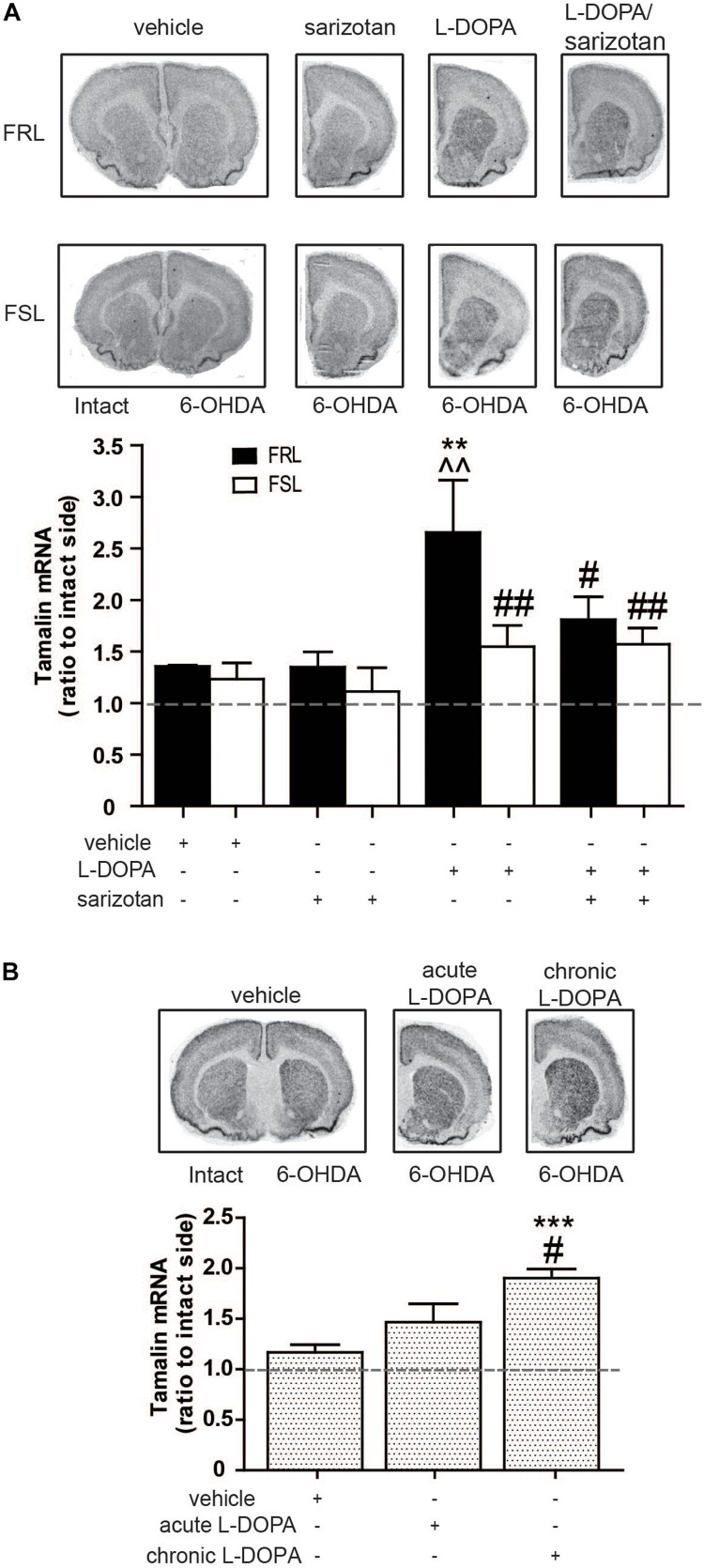
Chronic L-DOPA treatment increases in tamalin mRNA levels in unilaterally 6-OHDA-lesioned FRL and Sprague Dawley, but not FSL, rats. Autoradiograms and histograms of *in situ* hybridization experiment against tamalin mRNA in the striatum of **(A)** FRL and FSL rats chronically treated with vehicle, sarizotan (2.5 mg/kg, i.p.), L-DOPA (10 mg/kg, i.p.), and sarizotan/L-DOPA (2.5/10 mg/kg, i.p.) and **(B)** Spraque Dawley rats treated with vehicle, acute and chronic L-DOPA (10 mg/kg, i.p.). Data represent mean ± SEM of the ratio between the lesioned and the intact hemisphere for three to eight animals per group. (A)**p < 0.01 vs correspondent vehicle-treated group, ^^*p* < 0.01 vs correspondent sarizotan-treated group, ^#^*p* < 0.05, ^##^*p* < 0.01 vs FRL group chronically treated with L-DOPA, accordingly to two-way ANOVA followed by Fisher *post hoc* test; **(B)** ***p < 0.001 vs vehicle-treated group, ^#^*p* < 0.05 vs the group acutely treated with L-DOPA.

Within the list of genes that were found through the microarray analysis, tamalin, was chosen for further analyses with *in situ* hybridization although it has never been linked to AIMs before. However, the important role of tamalin in controlling the function of mGluR5 (Sugi et al., 2007) attracted our attention. Significant effects of genotype (*F*_1_,_25_ = 6.56; *p* < 0.05) and treatment (*F*_3_,_25_ = 5.69; *p* < 0.01) were found. *Post hoc* test showed that chronic treatment with L-DOPA increased (*p* < 0.01) tamalin mRNA in FRL but not FSL rats when compared to vehicle- or sarizotan-treated groups (Figure 11A). Moreover, sarizotan/L-DOPA-treated FRL rats had a significantly lower tamalin mRNA level than L-DOPA-treated FRL (*p* < 0.05; Figure 11A).

To confirm that tamalin expression gene is paralleled by L-DOPA responses not only in FRL rats, tamalin mRNA was measured also in unilaterally 6-OHDA-lesioned Sprague Dawley rats treated with vehicle, or with acute or chronic (4 weeks) L-DOPA. An overall significant treatment effect was found (*F*_2_,_15_ = 14.0; *p* < 0.001). *Post hoc* test showed that rats chronically treated with L-DOPA had a significantly higher tamalin mRNA both when compared to rats treated with vehicle and acute L-DOPA (*p* < 0.001 and *p* < 0.05, respectively; Figure 11B).

## Discussion

Depressed patients have a two-fold increased risk to develop PD when compared to healthy siblings and the general population. Tacrine induces jaw movements in rodents that have been suggested to model parkinsonian tremor (Salamone et al., 1998). FSL rats were found to be more responsive to tacrine-induced tremorous jaw movements which is consistent with their supersensitivity toward cholinergic compounds (Overstreet et al., 2005). In a clinical perspective it is interesting to note that both parkinsonian tremor and depressive symptomatology can be counteracted by cholinergic muscarine receptor antagonists (Drevets et al., 2013; Kalia and Lang, 2015). A speculation derived from the data on tacrine actions in FSL rats is that precautions should be taken to prescribe cholinesterase inhibitors to depressed parkinsonian patients with a tremor-dominant type. Vice versa, such patients may have antitremor as well as antidepressant responses to anti-cholinergic compounds. Our data also suggest that a depression-like genetic background combined with hemiparkinsonism results in a reduced response to antiparkinson treatments compared to control “non-depressed” FRL rats. Thus, chronic treatment with L-DOPA caused development of supersensitization and dyskinetic behaviors in FRL, but not in FSL, rats. To our knowledge, hemiparkinsonian FSL rats represent the first rat strain that have reduced AIMs upon chronic L-DOPA treatment. In the clinical setting, it is known that patients which develops supersentization and dyskinesia toward L-DOPA have significant problems with depression and anxiety (van der Velden et al., 2018). However, to our knowledge, it remains to be studied whether parkinsonian patients with depression are more likely to develop L-DOPA supersensitization and dyskinesia when compared to matched patients without depression. Effects of the antidyskinetic agent sarizotan were attenuated in FSL rats. Co-administration of sarizotan and L-DOPA prevented dyskinesia, both in terms of sensitization to turning and AIMs, in FRL, but not FSL, rats. A possible explanation is the finding of decreased levels of 5-HT_1__A_ receptor in the raphe nuclei of FSL rats chronically treated with L-DOPA.

The reduced L-DOPA response in FSL rats provides an interesting opportunity to learn more about molecular mechanisms underlying the development of L-DOPA-induced sensitization and AIMs. For this reason, a series of experiments were performed to identify parallels between gene expression markers previously linked to dyskinesia and the dramatic different behavioral response found in the hemiparkinsonian FSL and FRL rats. As expected, we found that striatal c-fos mRNA was increased in FSL, compared to FRL, rats. We did not find any genotype difference to an acute challenge with Apomorphine, a D_1_/D_2_ receptor agonist, or L-DOPA. These data, together with the previous finding that D_1_ receptors are actually increased in FSL rats (Bjørnebekk et al., 2007) argue against a prominent role of alterations in the dopaminergic system underlying the lack of supersentization toward L-DOPA in FSL rats. As mentioned above, several non-dopaminergic systems have been implicated in L-DOPA-induced dyskinesias and we studied several of them. No parallels between the behavioral responses and the levels of striatal opioid neuropeptides or serotonin markers were found. This discrepancy could be due to several contributes. Importantly, FRL and FSL rats are selectively bred strains and differ from Sprague Dawley rats which are normally used in preclinical studies of L-DOPA induced dyskinesia. Moreover, our data could further indicate that the interval between the last administration of L-DOPA and the time of death has to be taken into account when interpreting results on the level of opioid peptides (Sgroi and Tonini, 2018). Furthermore, there are contradictory reports when it comes to alterations in SERT in dyskinetic PD patients (Kish et al., 2008; Rylander et al., 2010).

Since the difference between the FRL and FSL rats in response to L-DOPA did not correlate to changes in opioid peptidergic or serotonergic correlates of dyskinesia, we decided to search for other biochemical correlates to elucidate this difference. We therefore performed an exploratory microarray experiment with the striata of FRL and FSL animals chronically treated with L-DOPA and found a parallel behavioral responses and c-fos [along with other Fos/Jun members including fosB (Supplementary Table S3)]. Moreover, a correlation between increased vGAT and behavioral responses was also found. An alteration of vesicular GABA release in L-DOPA-treated animals was previously reported (Wang et al., 2007) and our result further supports a role of the GABAergic system in the modulation of L-DOPA supersentization and dyskinesia.

An additional novel finding is the parallel alterations between an increased tamalin mRNA expression and L-DOPA-induced sensitization and AIMs. Tamalin is a scaffold protein highly expressed in the brain that interacts with metabotropic groups 1 and 2 glutamate receptors and GABA_B__2_ receptors along with specific guanine nucleotide exchange factors (Nevrivy et al., 2000; Sugi et al., 2007). Tamalin has been shown to have an autoinhibiting conformation when its concentration is low and when the concentration increases, the binding with mGluR5 receptors stabilizes the active conformation (Sugi et al., 2007). mGluR5 receptors are critically implicated in the development of L-dopa-induced dyskinesias (Sgambato-Faure and Cenci, 2012). Tamalin also interacts with scaffolding proteins, including PSD-95, S-SCAM, SAPAP1/3, Mint2, and CASK, that are involved in postsynaptic organization and protein trafficking in neuronal cells. Thus, through these multiple interactions, tamalin is positioned to control a variety of signaling pathways that could be involved in the mediation of AIMs. In this context, it is intriguing that mGluR5 receptors are reduced in FSL rats (Kovačević et al., 2012). It is possible that a reduced upregulation of mGluR5/Tamalin signaling contributes to the reduced L-DOPA responses in FSL rats.

In conclusion, our study suggests that a genetic depression-like rat model have altered behavioral and transcriptional responses in experimental parkinsonism and to antiparkinson drugs. Several adaptations in non-dopaminergic systems appears to underlie this differential responsivity and, in particular, induction of the mGluR5 adaptor protein, tamalin, is a novel correlate to L-DOPA-induced supersentization and dyskinesia.

## Data Availability Statement

The data generated for this study can be found in NCBI using the accession numbers MN474033–MN475147.

## Ethics Statement

The experiments were performed in agreement with the European Communities Council Directive of 24 November 1986 (86/609/EEC) on the ethical use of animals and were approved by the local Ethical Committee at Karolinska Institutet.

## Author Contributions

NSc performed the research, analyzed the data, and wrote and edited the manuscript. XZ performed the research, analyzed the data, and edited the manuscript. NSt analyzed the data and edited the manuscript. AM contributed with essential reagents and edited the manuscript. PA edited the manuscript. PS designed the study and wrote the manuscript.

## Conflict of Interest

The authors declare that the research was conducted in the absence of any commercial or financial relationships that could be construed as a potential conflict of interest. The reviewer, GF, declared a shared affiliation, though no other collaboration, with several of the authors, PS, XZ, NS, and AM, to the handling Editor.

## Supplementary Material

TABLE S1 Primers used to generate riboprobes.

TABLE S2 Genes expressed in the 6-OHDA-lesioned striata of FRL and FSL chronically treated with vehicle. List of genes obtained from the microarray analysis of the RNA extracted from 6-OHDA-lesioned striata of FRL and FSL chronically treated with vehicle. Selecting criteria was made in the way that only genes that were found to be 1.5-fold statistically different at least in one experimental group from the vehicle-treated FRL group were listed.

TABLE S3 Genes expressed in the 6-OHDA-lesioned striata of FRL and FSL chronically treated with L-DOPA. List of genes obtained from the microarray analysis of the RNA extracted from 6-OHDA-lesioned striata of FRL and FSL chronically treated with L-DOPA/benserazide (10/7.5 mg/kg, i.p.). Selecting criteria was made in the way that only genes that were found to be 1.5-fold statistically different at least in one experimental group from the vehicle-treated FRL group were listed.
