# Supplementary material for: Non-dopaminergic Alterations in Depression-Like FSL Rats in Experimental Parkinsonism and L-DOPA Responses
Source: Front Pharmacol. 2020 Mar 20;11:304. doi: 10.3389/fphar.2020.00304 (PMC7099513; doi:10.3389/fphar.2020.00304)
Supplement: TABLE S1 — Primers used to generate riboprobes. [file Table_1.doc]

**Supp. Table 1**

*Primers used to generate riboprobes.*

| **Gene** | **accession N** | **fragment size** | **primer F** | **primer R** |
| --- | --- | --- | --- | --- |
| **vGAT** | NM_031782.1 | 435 bp | GGGAGACATTCATTATCAG | CTCACCACTACGTACAAGAT |
| **Tamalin** | NM_138894.1 | 455 bp | TTTACTCAGAGTCCTGAACA | GACTCCAGTGTGTCATAGAT |
